# Redox-immune modulating gadolinium vanadate based nanozymes for osteoarthritis therapy

**DOI:** 10.1016/j.mtbio.2026.103669

**Published:** 2026-09-15

**Authors:** Shitang Song, Wei Liu, Ningyi Guo, Siqi Wang, Yang Liu, Xi Gong, Dawei Li, Shanyue Guan, Bingbing Xu

**Affiliations:** a Sports Medicine Department, Beijing Key Laboratory of Sports Injuries, Peking University Third Hospital, Beijing, 100191, China; b Institute of Sports Medicine of Peking University, Beijing, 100191, China; c Department Sports Medicine and Exercise Rehabilitation, Beijing Sport University, Beijing, China; d Senior Department of Orthopedics, The Fourth Medical Center of PLA General Hospital, Beijing, 100091, China; e Technical Institute of Physics and Chemistry, Chinese Academy of Science, Beijing, 100190, China

**Keywords:** Osteoarthritis, Nanozyme, Pterostilbene, Cartilage protection, Antioxidant therapy

## Abstract

Osteoarthritis (OA) remains a highly prevalent degenerative joint disorder driven by a self-reinforcing cycle of reactive oxygen species (ROS) accumulation, inflammatory amplification, and chondrocyte dysfunction within the joint microenvironment. Herein, we developed a rare-earth (RE) doped-engineered gadolinium vanadate-based bioactive nanosystem through rational lanthanide modulation and pterostilbene loading for synergistic regulation of OA progression. Through precisely controlled RE lattice doping and electronic-structure optimization, GVCP exhibits potent enzyme-mimetic ROS-scavenging activity, thereby alleviating oxidative stress and suppressing inflammation in the pathological joint microenvironment. Beyond this upstream microenvironmental regulation, GVCP directly preserves chondrocyte homeostasis by attenuating apoptosis, accompanied by modulation of the p53 signaling pathway and restoration of the Bax/BCL-2 balance. Meanwhile, GVCP promotes macrophage polarization toward the reparative M2 phenotype, thereby contributing to immune remodeling favorable for cartilage protection. In addition, DFT calculations further reveal Re-doped-induced electron-transfer characteristics and catalytic energetics underlying the enhanced redox activity of the nanosystem. *In vivo* results further demonstrated that GVCP markedly mitigates cartilage degeneration, leading to effective attenuation of OA progression with good biocompatibility. As a result, this work proposes a bioactive nanotherapeutic strategy that combines upstream microenvironment regulation with downstream pathway intervention, offering a comprehensive antioxidant-immune modulation nanoplatform.

## Highlights

• Cerium doping enhances redox catalysis in gadolinium vanadate nanozymes.
• Redox regulation and pterostilbene delivery preserve chondrocyte homeostasis.
• Gadolinium contributes to reparative macrophage polarization.
• GVCP treatment preserves cartilage, improves mobility and relieves osteoarthritic pain.

## Graphical abstract

**Schematic diagram of the GVCP nanoplatform**: from rational design and fabrication to therapeutic performance *in vitro/vivo* and the underlying mechanisms.

**Figure:**
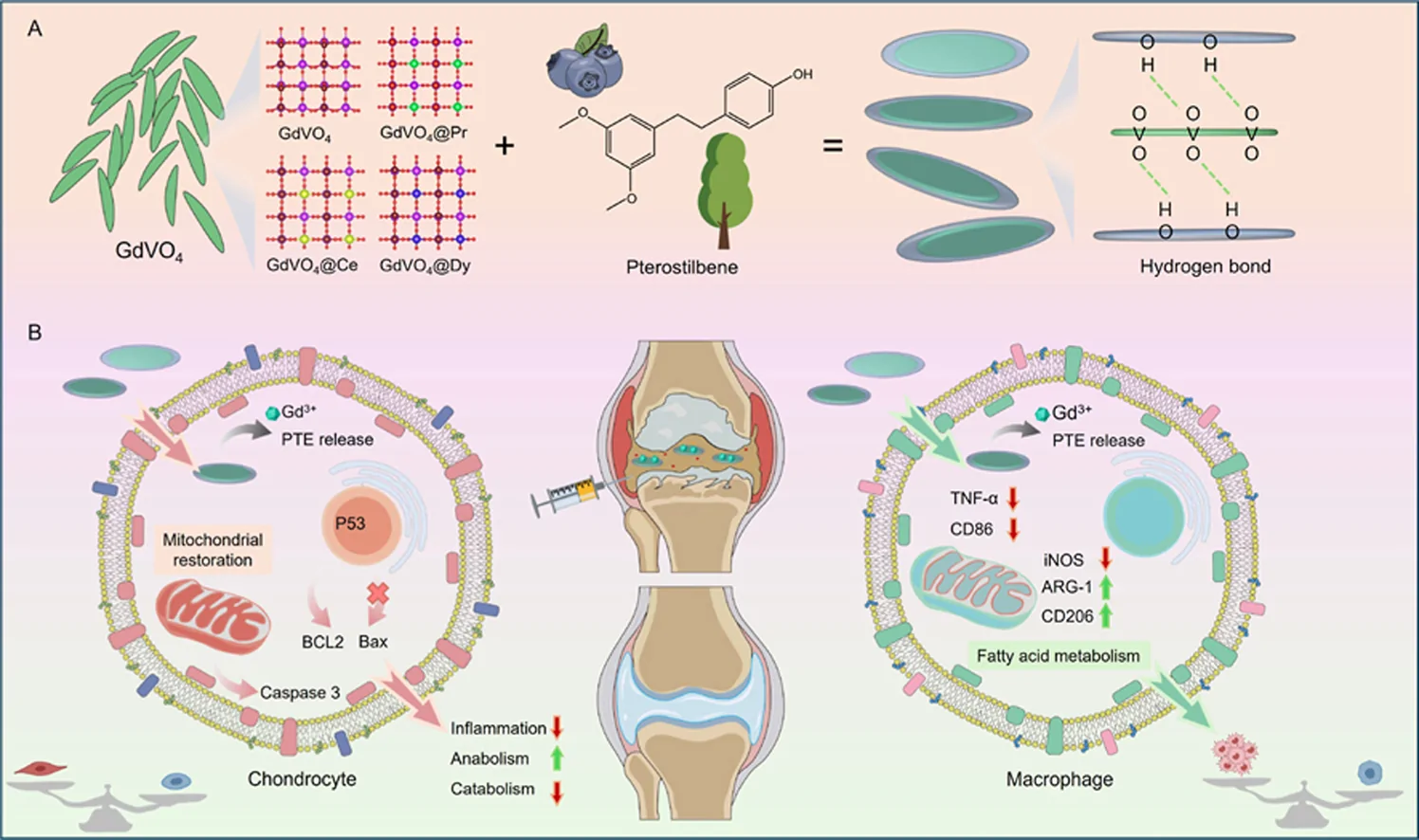

## 1 Introduction

Osteoarthritis (OA) stands as the most prevalent chronic degenerative joint disorder worldwide and profoundly compromises the quality of life for millions of patients [[1], [2], [3], [4]]. It is a major contributor to global disability [5], with considerable associated healthcare expenditures and socioeconomic impacts [6,7]. Although traditionally regarded as a mechanically driven disorder, OA is now widely recognized as a whole-joint disease involving persistent low-grade inflammation, progressive cartilage destruction, synovial dysfunction, subchondral bone remodeling, and osteophyte formation [[8], [9], [10], [11], [12], [13], [14], [15]]. Despite extensive efforts, no effective disease-modifying osteoarthritis drugs (DMOADs) have successfully translated into routine clinical practice [16,17]. Traditional treatments, like NSAIDs, steroids, and hyaluronic acid, mainly just ease the symptoms temporarily, rather than stopping the disease from getting worse [18]. In addition, their long-term use often leads to gastrointestinal and cardiovascular side effects [[19], [20], [21]]. Therefore, identifying key pathological mechanisms and developing effective new strategies remain urgent tasks in osteoarthritis research (see Scheme 1).

**Scheme 1:**
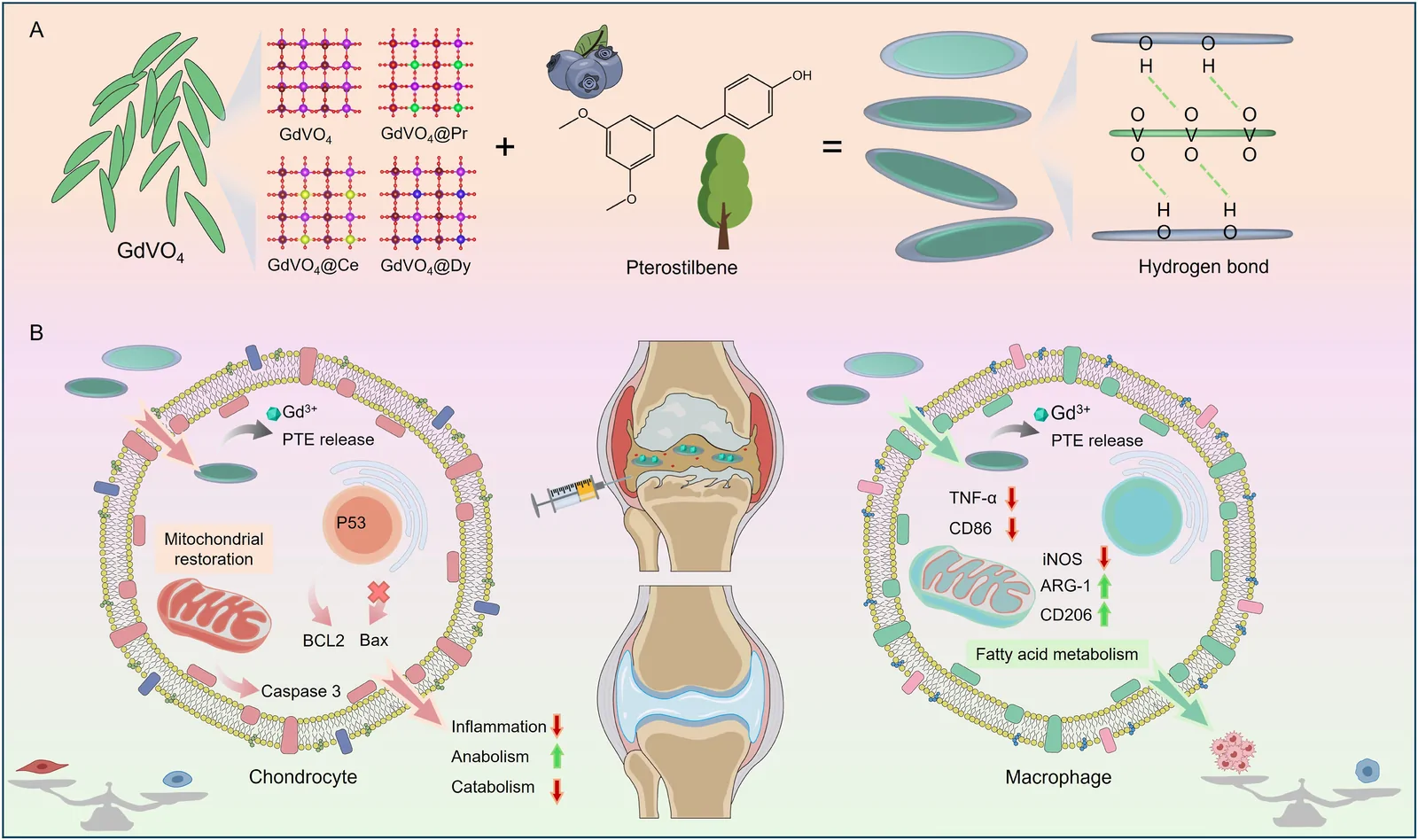
Preparation of GVCP and its proposed mechanisms for protecting chondrocytes and regulating macrophage polarization in osteoarthritis.

Recent studies into the etiology of OA have identified a self-perpetuating cycle between oxidative stress and inflammatory responses as the key driver of disease initiation and progression [[22], [23], [24]]. Within the OA-related microenvironment, mitochondrial dysfunction in chondrocytes, along with the overactivation of the NADPH oxidase (NOX) family and upregulation of nitric oxide synthase (NOS), leads to a large buildup of reactive oxygen species (ROS) and reactive nitrogen species (RNS), which disrupts cellular balance [[25], [26], [27], [28], [29], [30], [31], [32], [33], [34], [35]]. Meanwhile, excessive ROS disrupt the homeostasis of chondrocytes by inducing apoptosis, senescence, and impaired autophagic flux, which together accelerate matrix degradation and cartilage loss [[36], [37], [38]].

Although endogenous antioxidant enzymes such as SOD, CAT, and GPx help maintain redox balance under physiological conditions, their protective effects are often insufficient in the chronically inflamed OA joint [[39], [40], [41]]. Traditional antioxidants and bioactive small molecules have shown some promise, but their clinical potential is often limited by poor stability, low bioavailability, and rapid clearance [[42], [43], [44]]. As nanomaterials with intrinsic enzyme-like catalytic activity, nanozymes have become a promising platform for the continuous elimination of reactive oxygen species (ROS) due to their catalytic stability and tunable physicochemical properties [[45], [46], [47]]. Recent nanozyme-based OA therapies have demonstrated clear benefits in catalytic redox regulation, including single-atom nanozymes with SOD/CAT-like activity, zwitterionic nanozymes with antioxidant and hyaluronan-related functions, Mn_3_O_4_ nanozymes, and Cu-based MOF nanozymes [[48], [49], [50], [51], [52], [53]]. However, most reported systems mainly focus on ROS elimination or single-component functional design, while simultaneous regulation of oxidative stress, immune remodeling, and chondrocyte-protective signaling remains insufficiently explored.

To address this gap, we designed a rare-earth-based nanosystem that integrates catalytic antioxidant activity, immunomodulation, and bioactive molecule delivery. Gadolinium-containing materials have shown the ability to regulate macrophage behavior in addition to scavenging ROS [54,55], while pterostilbene (PTE) exhibits anti-inflammatory and cytoprotective activities through pathways related to Nrf2, PI3K/Akt, and NF-κB signaling [[56], [57], [58]]. Nevertheless, the therapeutic application of PTE is restricted by poor aqueous solubility and rapid metabolic elimination [54,59,60]. Based on these considerations, we constructed a Ce-engineered GdVO_4_ (GVC) nanozyme and further incorporated PTE to obtain a combined nanoplatform (GVCP). In this system, the Ce^3+^/Ce^4+^ redox pair provides enzyme-mimetic ROS-scavenging activity, the Gd-based matrix contributes to macrophage immunoregulation, and the nanocarrier improves the delivery performance of PTE. Using this platform, we investigated whether simultaneous regulation of oxidative stress, inflammation, and chondrocyte dysfunction could provide more effective intervention against OA progression. Our results indicate that GVCP protects chondrocytes from oxidative injury, reduces the expression of matrix-degrading enzymes, promotes macrophage polarization toward an anti-inflammatory phenotype, and alleviates cartilage degeneration and synovitis in vivo, supporting its potential as a bioactive nanotherapeutic strategy for OA treatment.

## 2 Results and discussion

### 2.1 Characterization and ROS scavenging performance of RE-doped GV

Initially, rare earth chlorides (RECl_3_, RE = Eu, Dy, Ce, Y) were dissolved in deionized water, then mixed with EDTA-2Na and Na_3_VO_4_ under stirring to synthesize RE-doped GV *via* a co-precipitation method. The resulting mixture was refluxed and dialyzed for 24 h each, followed by oven drying at 140 °C for 10 h. Finally, pterostilbene (PTE) was loaded onto the RE-doped GV nanoplatform (RE-doped GV@PTE). The RE-doped GV@PTE was first characterized by transmission electron microscopy (TEM). As shown in Fig. 1A–G, TEM analysis revealed a uniform fusiform morphology for both GV@Ce (denoted as GVC) and GV@Dy (denoted as GVD) with pure GV used as a control. The RE-doped GV nanoparticles exhibited a consistent particle size of ∼40–60 nm. High-resolution transmission electron microscopy (HRTEM) in Fig. 1C and G suggested clear lattice fringes with a spacing of 0.36 nm, assigned to the crystallographic plane of tetragonal GV. Notably, distinct lattice expansion was observed in the doped samples, confirming successful RE ion incorporation into the host lattice. Energy-dispersive X-ray spectroscopy (EDS) elemental mapping (Fig. 1D and H) verified the homogeneous distribution of Gd, Eu, V, O, Y, Ce/Dy elements throughout the GVC and GVD nanoparticles, further validating uniform RE doping. X-ray diffraction (XRD, Fig. 1I) was performed to confirm the crystal structure and RE incorporation. All samples exhibited characteristic diffraction peaks matching the (200), (112), and (312) planes of tetragonal GV (JCPDS #17-0260), demonstrating that the host crystal structure was well-preserved after RE doping. The diffraction peaks were moved to lower angel in the RE-doped samples, which unambiguously confirmed lattice expansion and effective substitution of the host lattice sites by RE ions, consistent with the HRTEM results. Electron paramagnetic resonance (EPR) spectroscopy was performed to evaluate the •OH scavenging capacity of the RE-doped GV, with the results shown in Fig. 1J. The control group exhibited strong characteristic EPR signals of •OH, while all RE-doped GV samples showed markedly reduced signal intensities, confirming the intrinsic •OH scavenging activity of the RE-doped nanosystems. Notably, the GVC group displayed the most dramatic attenuation of EPR signal intensity, with the lowest peak intensity among all doped samples, demonstrating that Ce doping endows the nanoplatform with the excellent •OH scavenging performance, outperforming the GV@Pr (GVP) and GVD. To evaluate the ROS scavenging activity of the RE-doped GV nanoplatforms, the methyl violet (MV) spectrophotometric assay was performed *via* UV-Vis absorption spectroscopy (Fig. 1K). MV can be specifically oxidized and degraded by ROS, resulting in a significant decrease in its characteristic absorption peak in the visible range (550–650 nm). The control group showed the lowest absorbance, indicating severe oxidative degradation of MV by excess ROS. In contrast, RE-doped GV samples partially preserved the MV absorbance by inhibiting •OH-mediated degradation. Consistent with the EPR results, GVC exhibited the highest residual MV absorbance among the doped samples and outperformed the CeO_2_ positive control under the tested conditions. These findings identified GVC as the most active RE-doped formulation and supported its selection for constructing the PTE-loaded GVCP nanoplatform.

**Fig. 1:**
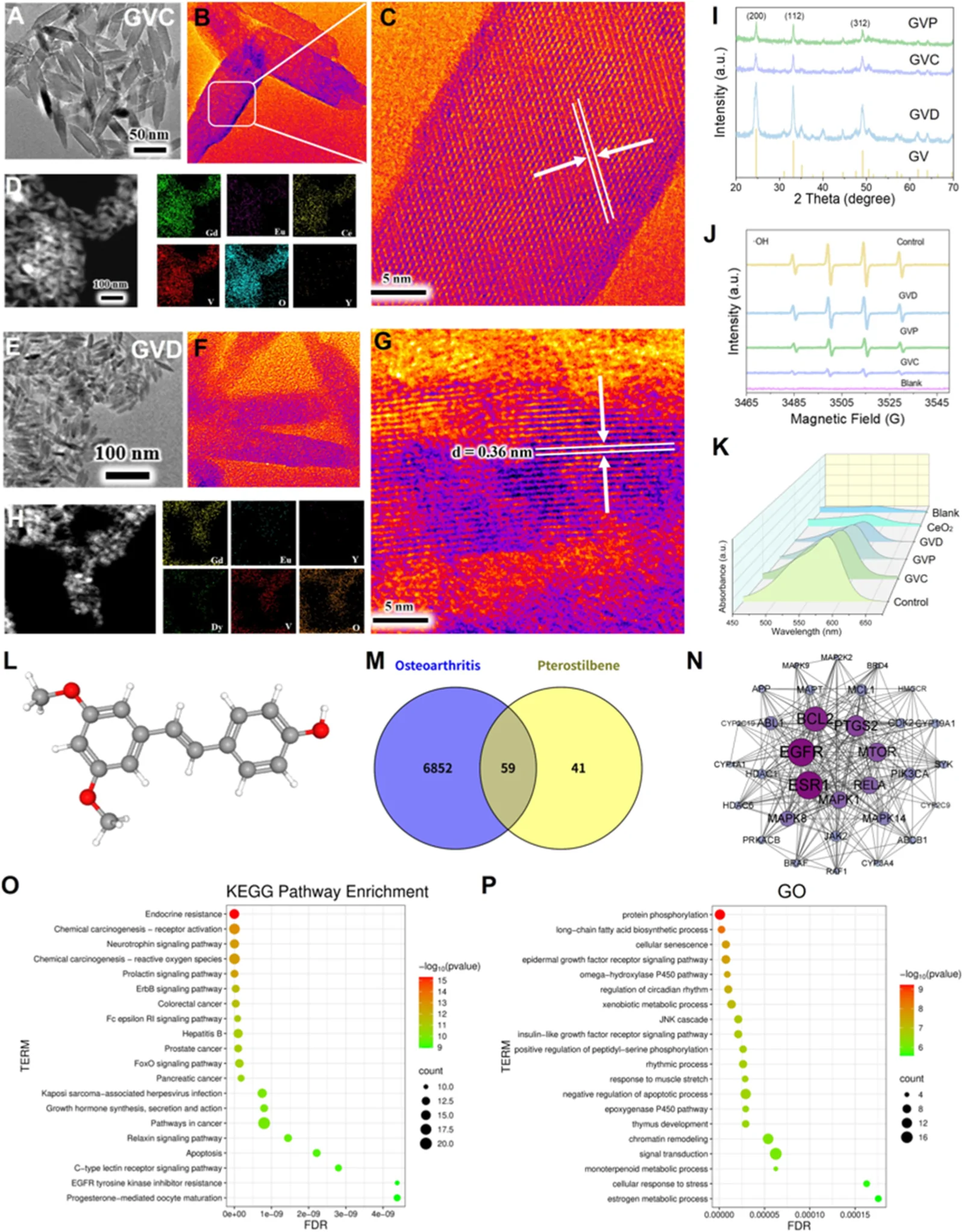
Characterization, ROS scavenging performance of RE-doped GV, and multi-target therapeutic mechanisms of pterostilbene against osteoarthritis. (A) HRTEM images, B) Pseudo-colored HRTEM image and C) lattice fringes with an interplanar spacing of GVC. (D) HAADF-TEM images and corresponding EDS elemental mapping of GVC. (E) HRTEM images, F) Pseudo-colored HRTEM image and G) lattice fringes with an interplanar spacing of GVD. (H) HAADF-TEM images and corresponding EDS elemental mapping of GVD. (I) XRD patterns of RE-doped GV samples. (J) EPR spectra for ROS scavenging evaluation. (K) UV-Vis absorption spectra of the MV assay for quantitative ROS scavenging assessment. (L) Chemical structure of Pterostilbene, a demethylated analog of resveratrol characterized by enhanced bioavailability and potent anti-inflammatory properties. (M) Venn diagram identifying 59 shared potential targets between Pterostilbene and Osteoarthritis associated gene sets. (N) PPI network analysis illustrating the interconnectedness of shared targets, with core hub genes such as EGFR, Bcl2, PTGS2, and MTOR highlighting key regulatory nodes. (O) KEGG pathway enrichment analysis revealing critical signaling cascades modulated by Pterostilbene, including Apoptosis, FoxO signaling, and EGFR tyrosine kinase inhibitor resistance pathways. (P) Gene Ontology (GO) biological process enrichment analysis emphasizing the role of Pterostilbene in regulating protein phosphorylation, signal transduction, and the apoptotic process within the articular microenvironment.

To further define the species-specific antioxidant profile of GVCP, we measured the SOD-like and CAT-like activities of GVCP and CeO_2_, respectively. GVCP showed higher SOD-like activity than CeO_2_, indicating a good capacity for superoxide removal (O_2_^−^; Fig. S1A). GVCP also exhibited higher CAT-like activity compared with CeO_2_ (Fig. S1B). Furthermore, OA pathogenesis is multifaceted, extending beyond oxidative stress to immune dysregulation and aberrant signaling network rewiring. To address this, we integrated the bioactive molecule PTE (Fig. 1L) into our GVC nanozyme scaffold to fabricate the GVCP composite platform. This design achieves strategic fusion of inorganic catalytic activity and organic pharmacological modulation, enabling dual-modal intervention targeting both the articular microenvironment and intracellular signaling cascades. Network pharmacology was employed to delineate the molecular mechanism of PTE, identifying 59 core pharmacodynamic targets (Fig. 1M) that underpin its multi-pathway chondroprotective effects. PPI network topological analysis (Fig. 1N) revealed BCL2, PTGS2 and MAPK family members as central hub nodes; these hubs are both direct PTE targets and exquisitely redox-sensitive, providing the molecular basis for GVC-PTE synergy. KEGG and GO enrichment analyses (Fig. 1O-P) further supported the combinatorial therapeutic advantages of GVCP: GVC scavenges ROS to mitigate p53-activating pathological stimuli, while PTE modulates downstream effectors to inhibit chondrocyte apoptosis and senescence.

This coupled “microenvironment restoration and signaling repair” mode of action confers robust synergistic efficacy for GVCP in alleviating synovial inflammation and remodeling the joint immune microenvironment. Collectively, our GVCP platform leverages the crosstalk between nanozyme catalysis and pharmaceutical modulation to break the oxidative damage-inflammatory amplification vicious cycle in OA, providing a sophisticated paradigm for multi-dimensional OA management.

### 2.2 Unveiling RE doping engineering mediated electronic state modulation and nanozymatic catalytic mechanism

To elucidate the mechanism by which RE doping regulates the SOD-like activity of GV, we constructed geometry optimized models of pristine GV and RE doped GV (*RE* = Ce, Pr, Dy) by isomorphous substituting Gd^3+^ lattice sites with Ce^3+^, Pr^3+^, and Dy^3+^ (Fig. 2A). Systematic first principles density functional theory (DFT) calculations were performed. Charge density difference analysis revealed significant local charge redistribution around the doping sites after RE incorporation (Fig. 2B), originating from strong orbital hybridization between the 4f orbitals of the dopant ions and the O 2p and V 3d orbitals of the lattice. Among the systems, GVC exhibited the most pronounced charge transfer, with notable charge depletion at the Ce site and charge accumulation at the coordinating O sites. Compared with Pr and Dy doped GV, GVC showed a greater degree of electron cloud shift between Ce and its coordinating oxygen, confirming the stronger valence adjustability and electron accepting/donating capability of Ce^3+^, which facilitates reversible valence transitions (Ce^3+^ ↔ Ce^4+^). This strong charge transfer and flexible valence fluctuation enable the Ce doping site to efficiently capture and activate ROS through rapid single electron transfer, providing a structural and electronic foundation for subsequent catalytic dismutation. Such localized charge redistribution not only optimizes the electron donor–acceptor characteristics of active sites but also establishes efficient electron transfer pathways for the catalytic cycle. Band structure calculations indicated that pristine GV has a wide bandgap, which hinders carrier migration and electron transitions during catalysis. In contrast, doping with Ce, Pr, or Dy introduced localized intermediate energy levels within the bandgap *via* characteristic RE 4f orbitals, significantly narrowing the apparent bandgap and enhancing carrier concentration and charge transport efficiency. This provides a thermodynamic basis for the continuous electron transfer processes required in SOD-like catalysis (Fig. 2C). Partial density of states (PDOS) analysis further elucidated the nature of this electronic structure modulation: the valence band maximum of pristine GV is primarily composed of O 2p orbitals, while the conduction band minimum mainly arises from V 3d orbitals (Fig. 2D). Upon RE doping, RE 4f orbitals contributed pronounced density of states peaks near the Fermi level. Notably, the 4f band center of Ce was closest to the Fermi level, corroborating its electron accepting/donating capability inferred from charge density difference analysis. This further explains, from an electronic structure perspective, the flexible valence transition behavior of Ce, which aligns well with the reversible single electron cycles required for superoxide anion dismutation. While Pr and Dy doping also optimized the orbital energy distribution of GV through 4f orbital modulation, enhancing superoxide radical capture and electron transfer efficiency, their effects were less pronounced than those of Ce doping. Based on these analyses, we calculated the Gibbs free energy profiles for the SOD-like catalytic reaction (Fig. 2E and F). The results showed that pristine GV exhibits a high energy barrier in the rate determining step, whereas RE doping significantly optimizes the adsorption free energy of reaction intermediates and reduces the energy barrier. Among all doped samples, GVC displayed the most favorable catalytic thermodynamics, featuring the lowest rate determining step barrier and moderate adsorption strength toward reaction intermediates. This balance avoids active site poisoning due to overly strong adsorption while maintaining sufficient driving force for the reaction, consistent with the electronic structure analysis and experimentally observed optimal SOD-like activity. In summary, the unique 4f electronic structure and variable valence states of RE ions enable precise modulation of the electronic structure and active site properties of GV through orbital hybridization, bandgap engineering, and energy level regulation. In particular, Ce doping achieves the most significant enhancement of SOD-like activity by virtue of its superior charge transfer capability, valence adjustability, and intermediate adsorption behavior. In conclusion, the present DFT analysis provides a theoretical rationale for the Ce-doping-associated catalytic behavior of the studied system.

**Fig. 2:**
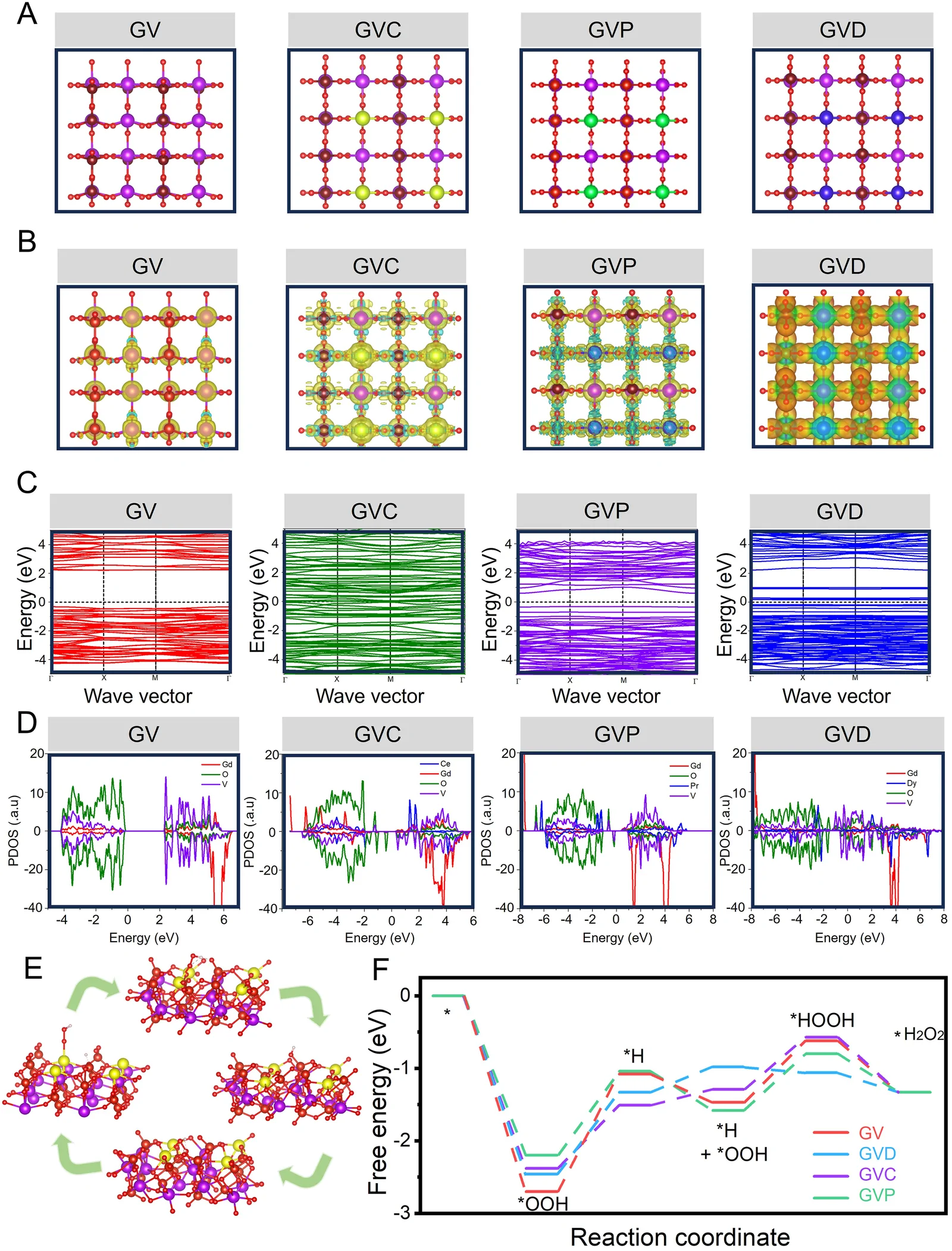
**Electronic structures and catalytic mechanisms of *RE* doped GV nanozymes.** (A) Optimized crystal structures of pristine *RE* doped GV, where purple, red, brown, yellow, green, and blue spheres represent Gd, O, V, Ce, Pr, and Dy atoms, respectively. (B) Electron density difference maps of the four systems, illustrating the charge redistribution induced by RE doping. (C) Calculated band structures, with the Fermi level set to 0 eV (dashed line), showing the electronic structure modulation after doping. (D) Projected density of states (PDOS) plots, decomposing the electronic states into contributions from Gd, O, V, and dopant (Ce/Pr/Dy) orbitals. (E) Schematic of the SOD-like activity pathway on the catalyst surface. (F) Free energy diagrams for the SOD-like activity on GV and *RE* doped GV (*RE* = Ce, Pr, Dy).

### 2.3 The ROS-scavenging and immunomodulatory capacity of GVCP

The development of OA is closely linked to chronic inflammation and oxidative stress. Within the joint microenvironment, inflammatory cascades worsen cartilage degeneration, leading to excessive production of reactive oxygen species (ROS) and chondrocyte apoptosis. This oxidative damage further disrupts cartilage metabolic balance, creating a harmful feedback loop that promotes osteoarthritis progression. Based on the ROS-scavenging property of GVC, this study aimed to further enhance its antioxidant function for better intervention in the progression of OA (Fig. 3A). To this end, PTE was selected as the active component to be loaded onto GVC, and Fourier transform infrared (FT-IR) spectroscopy was supplemented to verify the successful loading of PTE (Fig. S2). In a cell-free DPPH assay, GVCP exhibited the highest radical-scavenging activity, outperforming GVC, free PTE, and the GVC/PTE physical mixture (Fig. S3). The amount of loaded PTE was quantified by HPLC, yielding a loading efficiency of 55.0%. To assess the biocompatibility of GVCP (GVC loaded with PTE), we first evaluated its effect on chondrocyte viability *via* Live/Dead staining (Fig. S4). The results indicated that the nanoplatform possesses excellent cellular compatibility. Subsequently, intracellular ROS levels were evaluated using DCFH-DA fluorescence staining. Cells in the control group exhibited strong green fluorescence. In contrast, cells treated with GVCP and other treatments showed significantly reduced fluorescence intensity, with the GVCP-treated group displaying the weakest signal. These results suggest that GVCP can effectively scavenge ROS, thereby alleviating cellular oxidative stress (Fig. 3B and Fig. S5, *p* < 0.001). Beyond direct antioxidant activity, the immunomodulatory capacity of these formulations was evaluated using RAW264.7 macrophages. Stimulation with LPS successfully induced a pro-inflammatory M_1_ phenotype, characterized by the upregulation of CD86 (Fig. 3C and G) and iNOS (Fig. 3D and G). Intriguingly, the administration of GVCP markedly suppressed these M1 markers and pro-inflammatory cytokines, outperforming the monotherapies (Fig. S6, *p* < 0.01). Furthermore, when cells were polarized toward the anti-inflammatory M2 phenotype using IL-4, GVCP treatment further upregulated the expression of Arginase-1 (Arg-1, Fig. 3E and G), and CD206 (Fig. 3F and G). Subsequently, the immunomodulatory capacity of GVCP was further evaluated and compared with the GVC and PTE groups *via* flow cytometry. The results demonstrated that although GVC and GV individually exerted modest effects on macrophage behavior (Fig. S7), the GVCP exhibited significantly greater efficacy in promoting M2 macrophage polarization and exerting anti-inflammatory effects. Specifically, the proportion of CD206-positive cells in the GVCP group was markedly higher than that in the GVC and PTE treatment groups (Fig. 3H-I, *p* < 0.0001). Simultaneously, the expression of the M1 marker CD86 was most effectively downregulated in the GVCP group (Fig. 3J-K, *p* < 0.0001). Collectively, these data indicate that the integration of GVC and PTE exerts a synergistic regulatory effect, outperforming the individual components in remodeling the OA immune microenvironment. We speculated that the significant polarization of macrophages toward the M2 phenotype induced by GVCP was related to the release of Gd^3+^ from the system, a mechanism consistent with previously reported findings. To directly examine whether Gd^3+^ contributes to GVCP-induced macrophage polarization, we performed an additional Gd^3+^ chelation/control experiment. RAW264.7 macrophages were treated with control medium, GVCP, GVCP + DOTA, or GdCl_3_. Immunofluorescence staining showed that GVCP markedly increased the expression of the M2 markers CD206 and Arg-1 (Fig. S8). This effect was significantly reduced after Gd^3+^ chelation by DOTA, indicating that Gd^3+^ plays an important role in GVCP-mediated M2 polarization. In addition, GdCl3 alone partially increased CD206 and Arg-1 expression compared with the control group, further supporting the contribution of Gd^3+^ to macrophage repolarization. These results suggest that Gd^3+^ is involved in the immunomodulatory effect of GVCP, while the full M2-polarizing effect is achieved by the GVCP platform. Taken together, these findings confirm that GVCP functions as a potent immunomodulatory agent (Fig. 3L), effectively reprogramming macrophages from a pro-inflammatory M1 phenotype toward a pro-healing M2 phenotype, thereby offering an innovative therapeutic strategy for OA management.

**Fig. 3:**
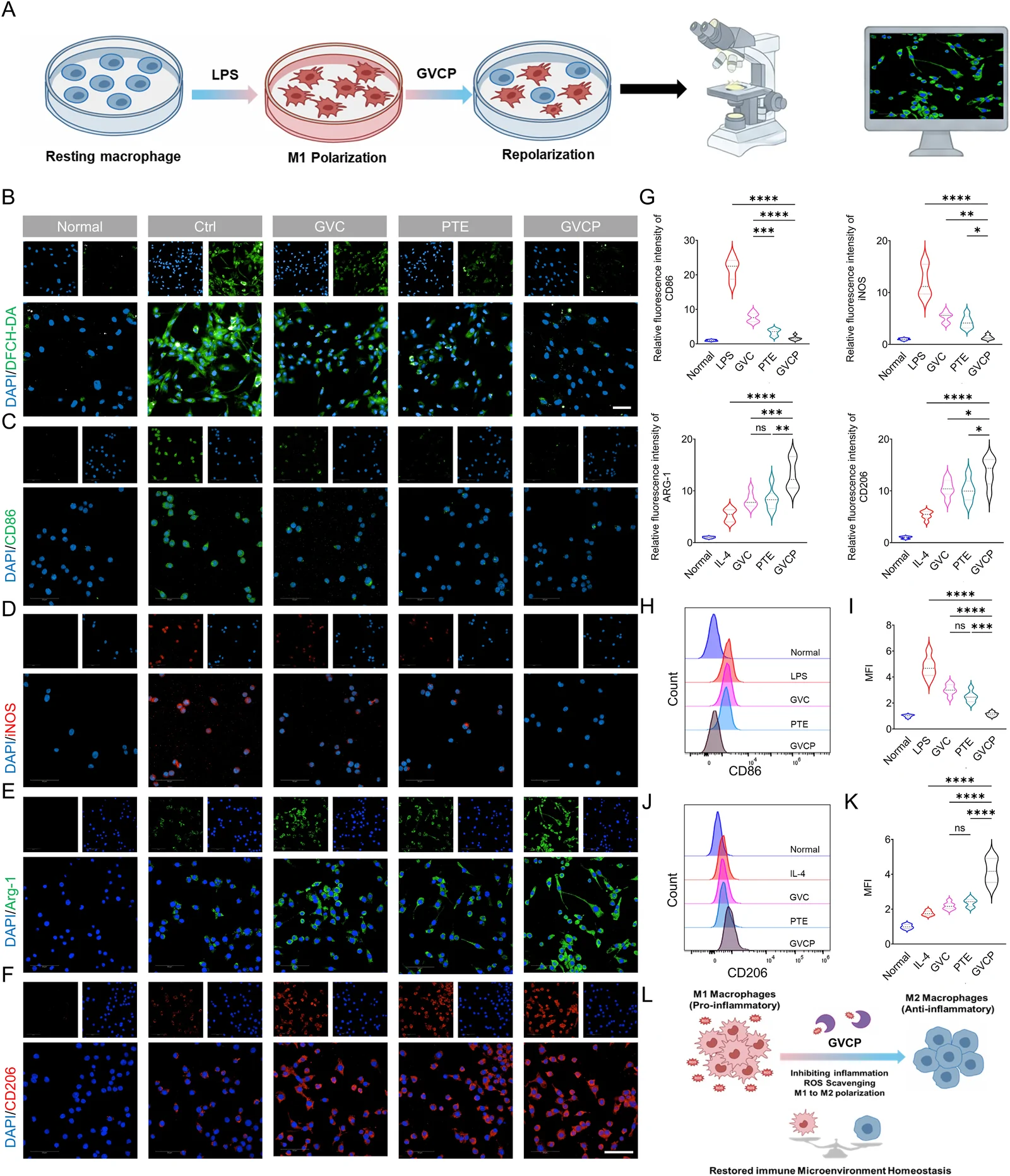
**Assessment of immunomodulatory capacity of GVCP.** A. Schematic illustration depicting the experimental workflow for cellular assays. B. Fluorescence imaging of ROS scavenging by GVCP. C-D. Representative immunofluorescence images of CD86, and iNOS. E-F. Representative immunofluorescence images of Arg-1, and CD206. G. The quantification of M1 or M2 fluorescence intensity. H-I. Flow cytometry analysis and fluorescence intensity analysis of CD206 expression in RAW264.7 cells. J-K. Flow cytometry analysis and fluorescence intensity analysis of CD86 expression in RAW264.7 cells. L. Schematic illustration of the mechanism by which GVCP reverses macrophage polarization. GVCP scavenges ROS *via* its nanozyme activity, thereby suppressing the inflammatory response and promoting the transition of macrophages from the pro-inflammatory M1 phenotype to the anti-inflammatory/reparative M2 phenotype. Scale bar: 50 μm. Data are presented as mean ± SD (n = 6). *p < 0.05, **p < 0.01, ***p < 0.001 and ****p < 0.0001 (One-way ANOVA with Tukey's post hoc for multi-group analysis).

### 2.4 The chondrocyte-protective capacity of GVCP

To evaluate the chondroprotective effect of GVCP, an *in vitro* OA model was created by treating primary chondrocytes with IL-1β, effectively recapitulating the catabolic and inflammatory milieu of the osteoarthritic joint (Fig. 4A). Immunofluorescence staining (IF) revealed that the expression of the cartilage matrix-degrading enzyme MMP-13 was significantly up-regulated under OA-like degenerative conditions (Ctrl group, Fig. 4B and G), while the expression of cartilage-specific markers (COL 2, SOX9, and ACAN) was markedly down-regulated (Fig. 4C–E, 4G and Fig. S9), accompanied by the activation of the apoptosis pathway (Fig. 4F and G and Fig. S10). In contrast, all treatment groups (GVC, PTE, and GVCP) significantly inhibited the expression of MMP-13 (Fig. 4B and G), restored the BCL-2/BAX ratio (Fig. 4F and Fig. S10), and thereby suppressed chondrocyte apoptosis, with the GVCP group exhibiting the strongest therapeutic effect (*p* < 0.05). These findings are highly concordant with our prior network pharmacology analysis (Fig. 1L–P), reinforcing the result that PTE-mediated OA mitigation is fundamentally anchored in the modulation of the BCL-2/BAX signaling cascade. At the mRNA level, qRT-PCR analysis further demonstrated that GVCP significantly up-regulated the expression of anabolism-related genes such as COL2A1, ACAN, and SOX9 in chondrocytes, while suppressing the expression of catabolism-related genes such as ADAMTS-5 and MMP13, thereby effectively halting chondrocyte degeneration (Fig. 4H, *p* < 0.05). The chondrocyte-protective effect was further validated by Western blot analysis, which revealed that GVCP treatment effectively rejuvenated the expression of chondrogenic proteins to near-physiological levels, substantially outperforming the GVC and PTE groups (Fig. S11A–11B, *p* < 0.05). To further examine whether the p53-Bax/BCL-2 axis is involved in GVCP-mediated chondroprotection, we performed a p53 gain-of-function experiment using the p53 activator C16-Ceramide (Fig. S12). IL-1β stimulation increased p53 and Bax expression and reduced BCL-2 expression in chondrocytes, indicating activation of apoptosis-related signaling. GVCP treatment suppressed p53 and Bax expression and restored BCL-2 expression. Notably, co-treatment with C16-Ceramide partially reversed these protective effects, as shown by higher p53 and Bax levels and lower BCL-2 expression compared with GVCP treatment alone. These findings provide functional evidence that GVCP protects chondrocytes by regulating the p53-Bax/BCL-2 apoptotic axis. Collectively, these results substantiate that GVCP exerts a multifaceted chondroprotective effect, thereby positioning this nanozyme-drug platform as a promising candidate for OA treatment.

**Fig. 4:**
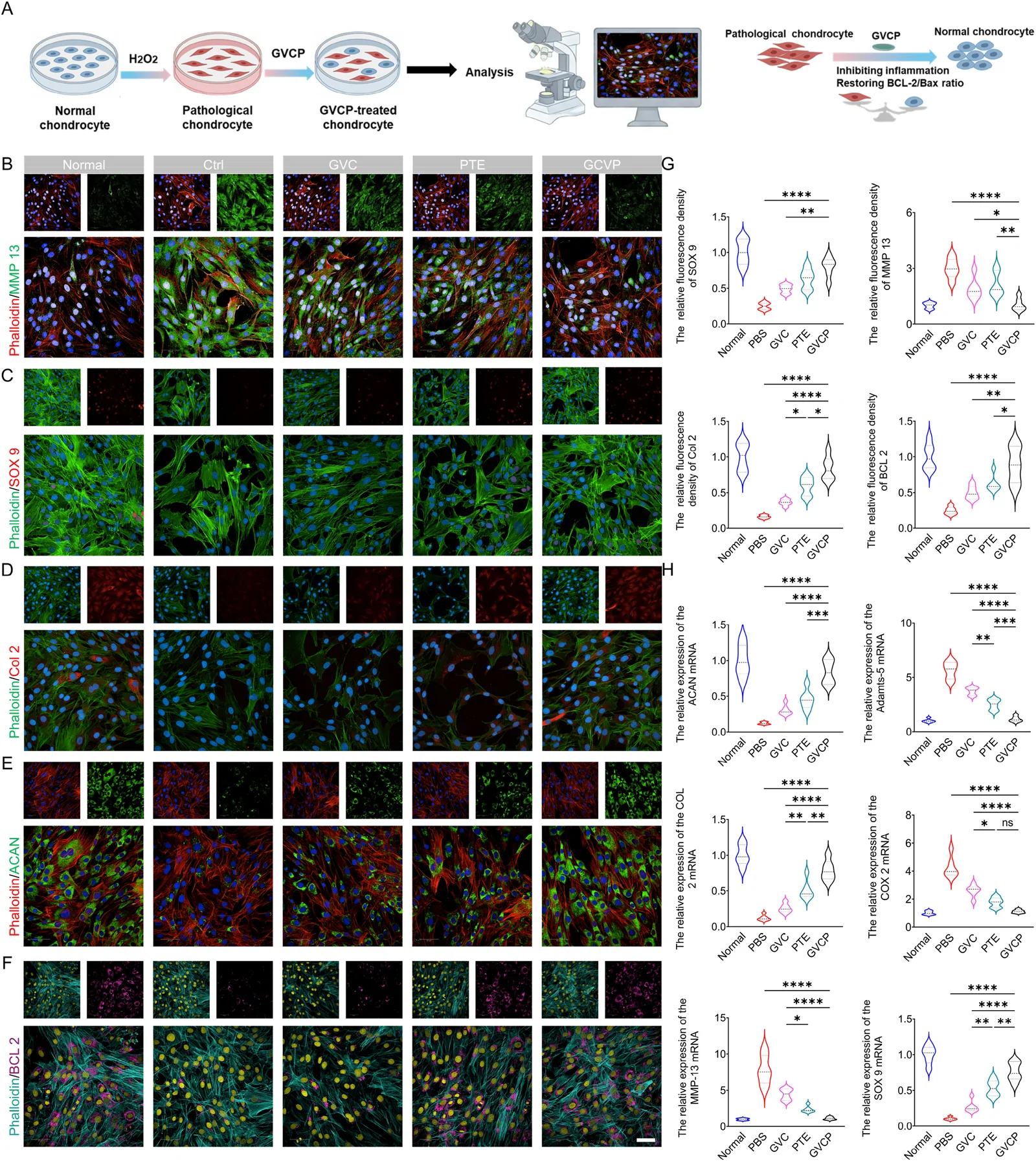
**The chondrocyte-protective effect of GVCP.** A. Schematic illustration depicting the experimental workflow for cellular assays. B-F. Representative IF images of MMP-13, COL 2, SOX9, ACAN and BCL-2 in primary chondrocytes. Scale bar: 50 μm. G. Quantitative analysis of MMP-13, COL 2, SOX9, and BCL-2 in primary chondrocytes. H. Relative mRNA expression levels of COL2A1, ACAN, ADAMTS-5, IL-1β, TNF-α, and MMP13. I. Representative western blot images of Adamts-5, COX-2, SOX9, BCL-2, BAX, MMP-13, COL 2, and ACAN in chondrocytes. GAPDH was used as a loading control. J. Quantitative analysis of Adamts-5, COX-2, SOX9, BCL-2, BAX, MMP-13, COL 2 and ACAN protein expression levels from (I). Data are presented as mean ± SD (n = 6). *p < 0.05, **p < 0.01, ***p < 0.001 and ****p < 0.0001 (One-way ANOVA with Tukey's post hoc for multi-group analysis).

### 2.5 GVCP significantly improves gait in osteoarthritic rats

The in vivo therapeutic effects of GVCP were systematically evaluated through a series of behavioral assessments, including gait analysis, rotarod tests, and hot plate tests, to assess functional recovery and pain relief. Quantitative gait analysis showed that rats in the PBS group exhibited significant motor function deficits, characterized by a reduced duty cycle (59% of the sham group), an increased front/rear print ratio (2.71-fold higher than sham), and a shortened stride length (52% of the sham group, Fig. 5A-B). Remarkably, the administration of GVCP effectively recalibrated these biomechanical metrics toward normal levels. Specifically, the duty cycle was restored from 41.54 ± 5.21 to 60.52 ± 6.75 (*p* < 0.05), while the stride length was nearly doubled (from 6.18 ± 0.29 to 11.27 ± 0.50 cm, *p* < 0.05), indicating a substantial alleviation of joint discomfort and mechanical dysfunction. While the GVC and PTE elicited marginal improvements, they failed to match the restorative effect of the GVCP treatment (Fig. 5B-C). The rotarod experiment was conducted in order to further assess the motor coordination and endurance frequently impaired by chronic osteoarthritis pain. The fall latency in the PBS group suffered a precipitous drop compared with the sham group (32.50 ± 5.63 s vs. 107.26 ± 10.89 s), reflecting severe proprioceptive impairment. Consistent with the gait data, GVCP treatment successfully rescued motor performance, extending the latency to 98.25 ± 13.33 s, a performance level markedly superior to those of the GVC (43.89 ± 4.47 s, *p* < 0.05) and PTE (63.54 ± 8.63 s) groups (Fig. 5D).

**Fig. 5:**
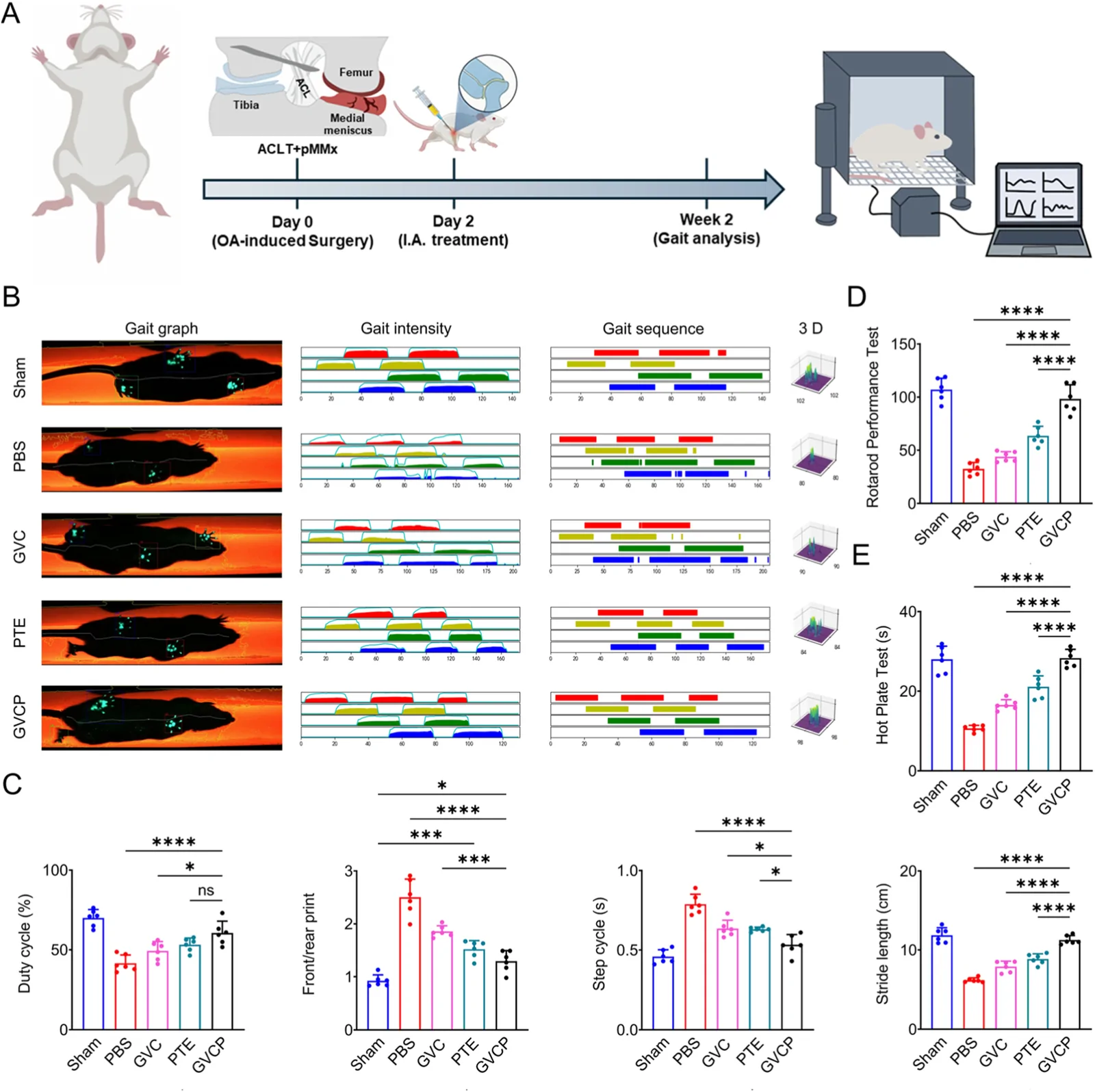
**Effects of intra-articular GVCP injection on gait performance in OA model rats.** A. Schematic illustration of the gait analysis in OA rat models. B. Representative illustrations of gait pattern, step sequence, and intensity. C. Comparative quantitative analysis of stride length, duty cycle, front/rear paw prints, and step cycle across the indicated groups. D. Quantitative analysis of the rotarod test among the groups. E. Quantitative analysis of the hot plate test among the groups. Data are presented as means ± SD (n = 6). *p < 0.05, **p < 0.01, ***p < 0.001 and ****p < 0.0001.

As a clinical hallmark of OA, the hypersensitivity state of injury sensitivity was then evaluated through the hot plate test. The PBS-treated group exhibited a significantly lower thermal withdrawal latency (10.64 ± 0.72 s), indicating a heightened state of hyperalgesia. GVCP treatment dramatically elevated the pain threshold, prolonging the response latency to 28.32 ± 2.15 s (*p* > 0.05 vs. sham, Fig. 5E). The analgesic potency of the GVCP system underscores its unique advantage in suppressing central or peripheral sensitization. Overall, these behavioral results confirm that the GVCP nanoplatform, through reactive oxygen species scavenging and anti-inflammatory mechanisms, not only alleviates cartilage degeneration but also achieves comprehensive restoration of motor function and relief of OA-related pain.

### 2.6 *In vivo* therapeutic efficacy

#### 2.6.1 GVCP inhibits osteophyte formation and restores subchondral bone

Encouraged by the significant functional recovery observed in the gait assays, we further investigated the impact of GVCP on the structural integrity of articular cartilage during osteoarthritis progression. The pathogenesis of late-stage OA is characterized by a deleterious crosstalk between cartilage degeneration and malaligned subchondral bone remodeling. To evaluate the effects of GVCP on osteophyte formation and subchondral bone sclerosis, micro-CT was employed to visualize the microarchitecture of the knee joint (Fig. 6A). Two-dimensional (2D) and three-dimensional (3D) micro-CT reconstructions revealed that PBS-treated joints suffered from extensive osteophyte proliferation and severe articular surface wear. Conversely, GVCP treatment substantially attenuated these pathological ossifications and mitigated subchondral sclerosis, demonstrating a therapeutic efficacy clearly superior to that of the GVC or PTE treatment (Fig. 6B). To further evaluate subchondral bone remodeling, we systematically analyzed the microstructural parameters of tibial subchondral bone, including Tb.Th, Tb.Pf, BV/TV, Tb.Sp, and Tb.N. As shown in Fig. 6C, all parameters in the GVCP group were significantly improved relative to the PBS group and approached the levels of the sham group. Specifically, Tb.Th increased from 0.083 ± 0.016 mm to 0.196 ± 0.033 mm (*p* < 0.0001), BV/TV rose from 27.0 ± 7.4% to 57.2 ± 9.3% (*p* < 0.001), Tb.N increased from 2.14 ± 0.56 mm^−1^ to 3.40 ± 0.54 mm^−1^(*p* < 0.05), Tb.Sp decreased from 0.263 ± 0.046 mm to 0.116 ± 0.016 mm (*p* < 0.05), and Tb.Pf reduced from 18.0 ± 2.0 mm^−1^ to 8.6 ± 1.2 mm^−1^(*p* < 0.001 vs. GVC). Notably, only GVCP effectively reversed these pathological changes, restoring the bone microstructural parameters to near-normal level. In contrast, the GVC and PTE groups showed only moderate improvement and failed to achieve the same restorative effect of GVCP. In addition, these cryo SEM/EDS results showed that the cartilage surface of PBS group was rough, accompanied by ectopic calcium/phosphorus deposition and a large loss of selenium (Fig. 6D). In contrast, the cartilage surface of GVCP group was smooth, and its element distribution was similar to that of sham operation group. In conclusion, these results indicate that GVCP not only significantly inhibits the formation of periarticular osteophytes and reduces subchondral osteosclerosis, but also promotes the normalization of articular cartilage surface morphology and elemental composition, thus effectively preventing the pathological progress of OA.

**Fig. 6:**
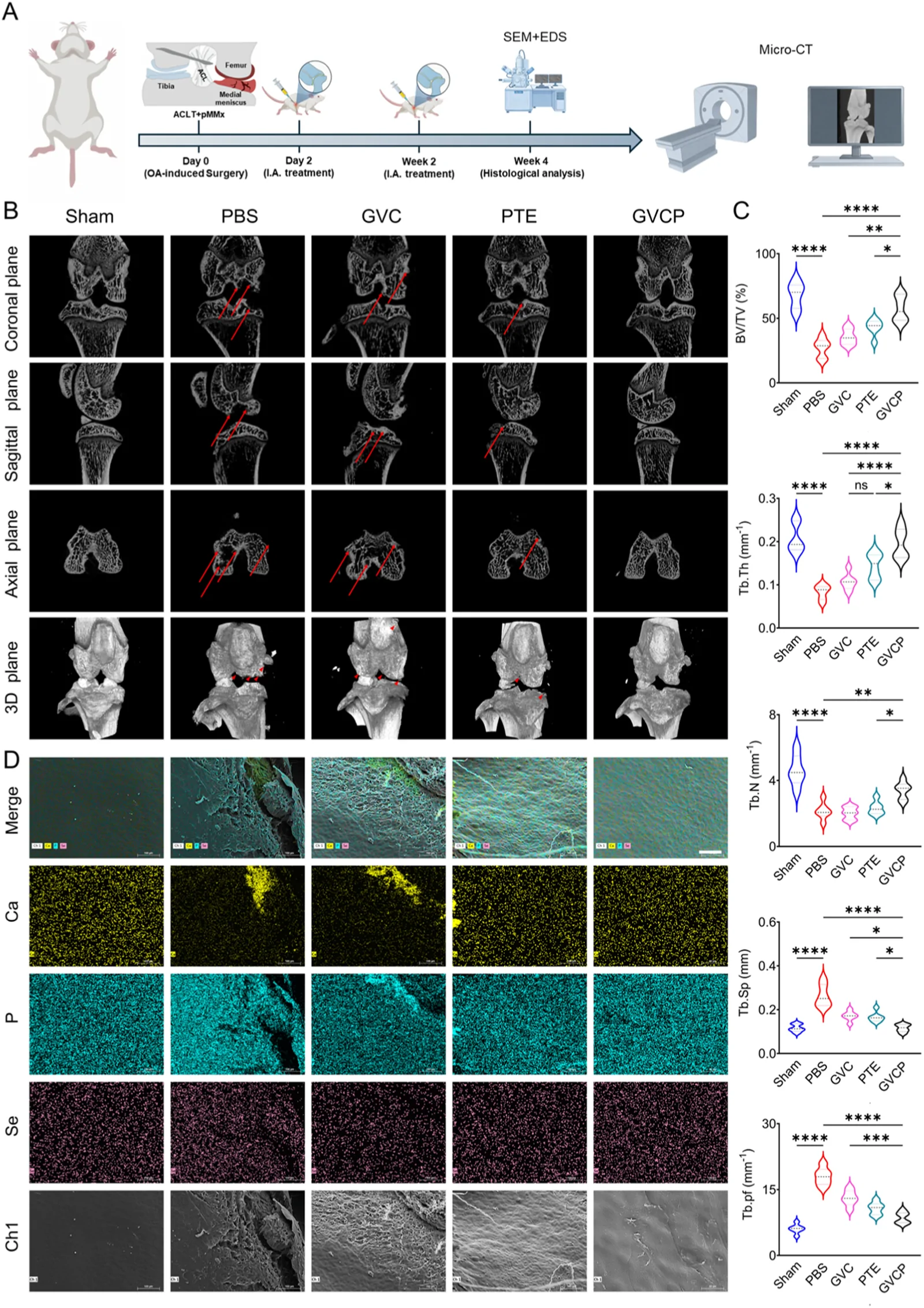
**GVCP reduces osteophyte formation and improves subchondral bone microarchitecture in osteoarthritic rats.** A. Schematic illustration of intra-articular treatment and subsequent analyses (SEM and micro-CT) in the ACLT + pMMx-induced OA rat model. B. representative micro-CT images showing 3D joint reconstructions (top) and 2D coronal slices (bottom), with osteophytes marked by red arrows. C. Quantitative micro-CT analysis of tibial subchondral bone parameters (Tb.Th, Tb.Pf, BV/TV, Tb.Sp, Tb.N; n = 3). D. Cryo-SEM/EDS characterization of cartilage surface and calcification (scale bar: 100 μm). Data are presented as means ± SD. *p < 0.05, **p < 0.01, ***p < 0.001 and ****p < 0.0001 (One-way ANOVA with Tukey's post hoc for multi-group analysis).

#### 2.6.2 GVCP for treating OA cartilage and synovium

To evaluate the therapeutic efficacy of GVCP, a systematic histopathological evaluation was performed using TB, SO&FG, and H&E staining (Fig. 7A–D). Histological results revealed that both the GVCP and sham groups maintained intact joint structure, which was characterized by smooth joint surface, uniform distribution of proteoglycans (homogeneous TB and SO staining, Fig. 7B-C), and normal synovial tissue structure and no inflammatory infiltration (Fig. 7D). In contrast, the PBS group exhibited severe osteoarthritic degeneration, articular cartilage fissure with a large amount of proteoglycan loss (faint TB and SO staining), synovial hyperplasia and thickening, and a large number of inflammatory cell infiltration. Quantitative analysis of cartilage degeneration using the OARSI scoring system further demonstrated that the OARSI score in the GVCP group (1.17 ± 1.17, *p* < 0.0001) was significantly lower than that in the PBS group (7.50 ± 1.05) and superior to those in the PTE (2.83 ± 0.75) and GVC (4.83 ± 0.75) groups (Fig. 7E). Similarly, the synovitis score in the GVCP group (0.50 ± 0.55, *p* < 0.05) was significantly reduced compared with the PBS group (3.67 ± 0.52) and was notably better than those in the GVC (2.67 ± 0.52) and PTE (1.50 ± 0.55 groups) (Fig. 7F). To further evaluate the durability of the therapeutic effect, we performed an additional 8-week assessment using micro-CT and histological staining (Figs. S13 and 14). At 8 weeks after surgery, the PBS group showed more severe joint degeneration, including obvious osteophyte formation, subchondral bone remodeling, cartilage surface damage, proteoglycan loss, and synovial inflammation. PTE or GVC monotherapy partially alleviated these pathological changes, but the protective effect was limited. In contrast, GVCP treatment maintained a smoother articular surface, reduced osteophyte formation and subchondral bone destruction, preserved proteoglycan staining, and attenuated synovial pathological changes. These 8-week results further support the sustained protective effect of GVCP against OA progression.

**Fig. 7:**
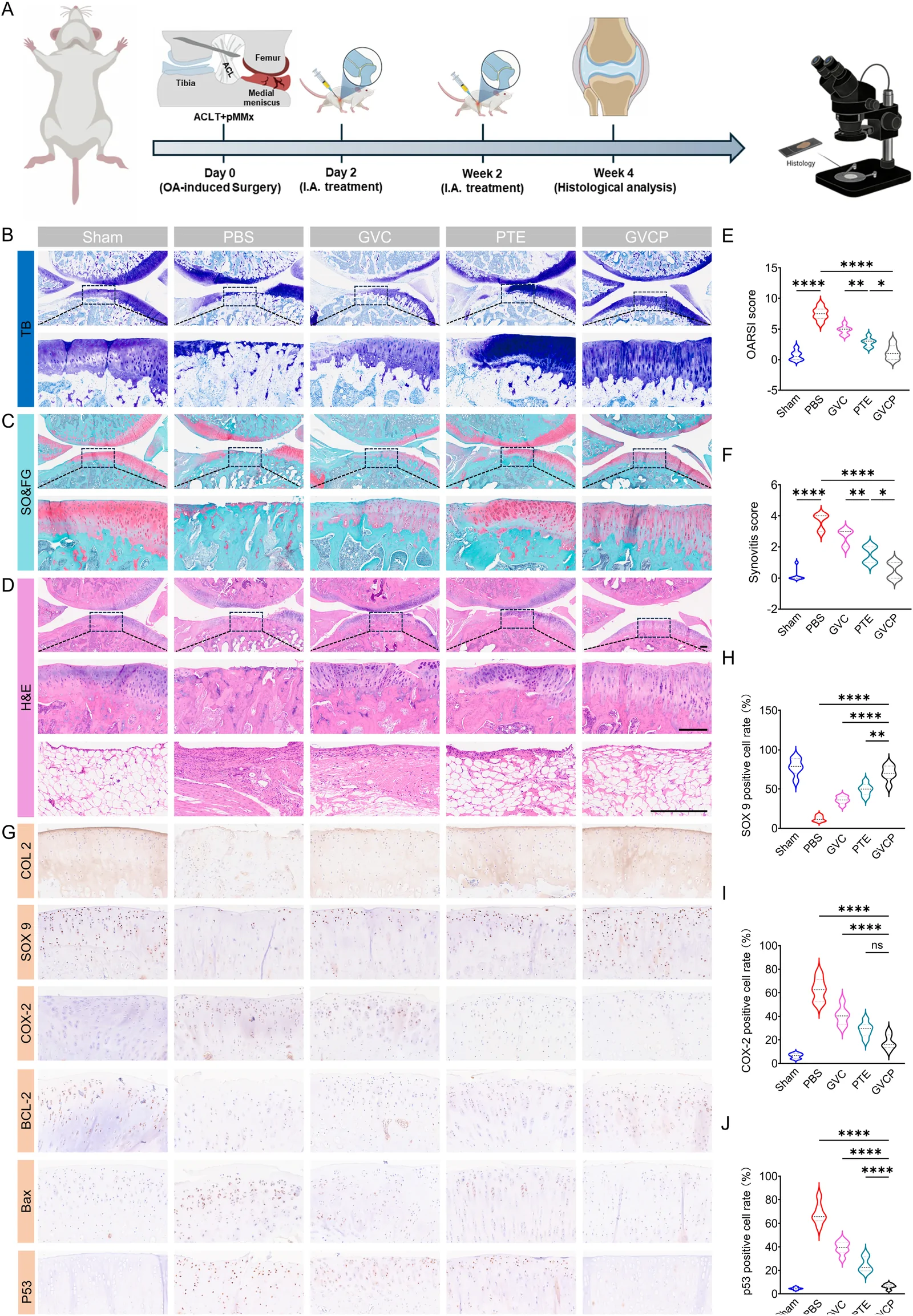
**GVCP protects articular cartilage and alleviates synovitis in osteoarthritic rats.** A. The schematic diagram of experimental workflow describes the timeline from in vivo intervention to subsequent pathological evaluation. B-D. Representative histological images of (B) TB, (C) SO&FG, and (D) H&E staining of knee joints from sham- or ACLT + pMMx-operated rats after 4-week treatment (scale bars: 200 μm). E-F. Corresponding semiquantitative scores for cartilage degeneration and synovitis. G. Representative IHC staining of SOX9, COL 2, COX-2, BCL-2, Bax, and p53 in articular cartilage (scale bars: 200 μm). H-J. Quantitative analysis of SOX9-, COX-2- and p53-positive chondrocytes in articular cartilage. Data are presented as mean ± SD (n = 6). *p < 0.05, **p < 0.01, ***p < 0.001 and ****p < 0.0001 (One-way ANOVA with Tukey's post hoc for multi-group analysis).

Then we used immunohistochemistry (IHC) to explore the protective effect of GVCP on articular cartilage degeneration at the molecular level. The expression of COL 2, the main structure of cartilage ECM, and Sox9, the main transcription factor of chondrogenesis, can be used as a healthy indicator of cartilage anabolism. IHC results showed that both GVCP group and sham group showed complete articular cartilage structure, COL 2 content was close to normal, and SOX9-positive chondrocytes were widely distributed (70.14 ± 9.54% vs. 78.33 ± 10.13% in the sham group, Fig. 7G-H). Conversely, the PBS group suffered a substantial loss of COL 2 and a massive reduction in SOX9 expression (12.33 ± 3.53%, Fig. 7H). Although GVC or PTE monotherapy showed partial ECM preservation, they failed to effectively halt cartilage degeneration, with SOX9-positive cell rates of 35.68 ± 4.73% (*p* < 0.0001) and 49.46 ± 7.26% (*p* < 0.01 vs. GVCP), respectively. These findings strongly confirm the combined effect of GVCP in inhibiting cartilage ECM degradation. Furthermore, GVCP treatment significantly downregulated COX-2 expression in chondrocytes, with the rate of positive cells decreasing from 62.53 ± 9.09% in the PBS group to 18.01 ± 5.56% (Fig. 7I). Given that COX-2 is the key rate-limiting enzyme catalyzing the synthesis of prostaglandin E2 (PGE2), these findings suggest that GVCP may alleviate OA-related pain by modulating the COX-2/PGE2 axis. Together with its inhibitory effects on inflammatory signaling pathways, these 4-week and 8-week results indicate that GVCP not only delays cartilage degeneration through anti-inflammatory and anti-apoptotic mechanisms but also provides sustained structural protection during OA progression.

Building on network pharmacology predictions, we confirmed that GVCP exerts therapeutic effects by modulating the p53-mediated apoptotic pathway. GVCP treatment dramatically decreased the population of p53-positive chondrocytes from 68.07 ± 8.93% (PBS group) to 6.50 ± 2.08% (Fig. 7G and J, *p* < 0.0001). Concurrently, it corrected the apoptotic balance by suppressing BAX and elevating BCL-2 expression. These findings confirm that inhibition of p53-driven apoptosis is the basic mechanism of GVCP-mediated OA protection (Fig. S15). In conclusion, GVCP shows strong therapeutic potential to delay the progression of OA by restoring the metabolic homeostasis of chondrocytes and inhibiting apoptotic signals.

#### 2.6.3 *In vivo* biosafety of GVCP for intra-articular administration

Finally, the in vivo biosafety of GVCP was evaluated after four weeks of intra-articular administration in ACLT + pMMx-induced OA rats. H&E staining of major organs, including the heart, liver, spleen, and kidneys, showed no obvious histological abnormalities in the GVCP-treated group compared with the sham group (Fig. S16). Consistently, complete blood count (CBC) parameters, together with hepatic and renal function biomarkers, remained within normal physiological ranges after GVCP treatment (Figures S17-18). These results indicate that intra-articular GVCP administration did not cause noticeable short-term systemic toxicity and exhibited good preliminary biocompatibility.

From a translational perspective, however, the in vivo fate of inorganic nanomaterials is closely associated with their particle size, surface chemistry, colloidal stability, and administration route [61,62]. Although intra-articular injection may reduce systemic exposure, joint retention and local clearance remain important factors that affect therapeutic durability in OA treatment [63]. In addition, previous studies on gadolinium-containing agents and gadolinium-based nanoparticles have emphasized the need to evaluate Gd distribution, tissue retention, clearance, and long-term safety during translational development [64]. Therefore, further studies are needed to clarify the joint-retention behavior, biodegradation of the GV-based scaffold, possible release of Gd/V species, long-term metabolic fate, and repeated-dose safety of GVCP.

## 3 Conclusion

In summary, we have developed a bioactive GVCP therapeutic nanoplatform that addresses the complex microenvironment of OA through the synergy of nanozyme-based ROS scavenging and drug delivery. Through the integration of DFT-supported catalytic activity and localized therapeutic release, this GVCP effectively halts disease progression. Molecular analysis demonstrates that GVCP protects chondrocytes by suppressing p53-mediated apoptotic cascades and balancing the BCL-2/Bax ratio. Furthermore, the pronounced polarization of macrophages toward the M2 phenotype induced by GVCP was found to be associated with the release of Gd^3+^ from the system, endowing the platform with the capacity to modulate the joint immune microenvironment. Collectively, this work establishes a promising therapeutic paradigm by synergizing nanozyme catalysis, targeted drug release and immune regulation, offering substantial translational potential for the clinical management of osteoarthritis and other degenerative joint disorders.

## CRediT authorship contribution statement

**Shitang Song:** Writing – original draft, Validation, Project administration, Data curation. **Wei Liu:** Visualization, Software, Investigation, Formal analysis. **Ningyi Guo:** Project administration, Methodology, Data curation. **Siqi Wang:** Software, Investigation, Formal analysis. **Yang Liu:** Supervision, Resources. **Xi Gong:** Supervision, Resources, Project administration. **Dawei Li:** Writing – review & editing, Investigation, Funding acquisition. **Shanyue Guan:** Writing – review & editing, Project administration, Methodology, Investigation, Formal analysis. **Bingbing Xu:** Writing – review & editing, Funding acquisition, Conceptualization.

## Declaration of competing interest

The authors declare that they have no known competing financial interests or personal relationships that could have appeared to influence the work reported in this paper.

## Data Availability

Data will be made available on request.

## Appendix A Supplementary data

Multimedia componenet 1

## Footnotes

Appendix A Supplementary data to this article can be found online at https://doi.org/10.1016/j.mtbio.2026.103669.
