# Supplementary material for: Redox-immune modulating gadolinium vanadate based nanozymes for osteoarthritis therapy
Source: Mater Today Bio. 2026 Sep 15;41:103669. doi: 10.1016/j.mtbio.2026.103669 (PMC13627812; doi:10.1016/j.mtbio.2026.103669)
Supplement: Supplementary file 1 [file mmc1.docx]

**Supplementary information**

**Redox-Immune Modulating Gadolinium Vanadate based Nanozymes for Osteoarthritis Therapy**

*Shitang Song^1,2 #^, Wei Liu^1,2#^, Ningyi Guo^1,2#^, Siqi Wang^3#^, Yang Liu^1,2^, Xi Gong^1,2^, Dawei Li^4*^, Shanyue Guan^5*^, and Bingbing Xu ^1,2*^*

1.Sports Medicine Department, Beijing Key Laboratory of Sports Injuries, Peking University Third Hospital, Beijing 100191, China.

2. Institute of Sports Medicine of Peking University, Beijing 100191, China.

3. Department Sports Medicine and Execise Rehabilitation, Beijing Sport University, Beijing, China.

4. Senior Department of Orthopedics, The Fourth Medical Center of PLA General Hospital, Beijing 100091, China.

5. Technical Institute of Physics and Chemistry, Chinese Academy of Science, Beijing 100190, P R. China.

Corresponding author:

Email: guanshanyue@mail.ipc.ac.cn

Email: xubingbing@hsc.pku.edu.cn

Email: ldw309@126.com

**Method and Material**

**Synthesis of GdVO_4_**

NaOH, GdCl_3_, EDTA-2Na and NaVO_3_ solutions were prepared separately. The NaVO_3_ aqueous solution was adjusted to pH~13 with 1 mol/L NaOH. Then 22 mL GdCl_3_, 16.5 mL EDTA-2Na and 16.5 mL NaVO_3_ solutions were added into a round-bottomed flask, vigorously stirred for 10 min to obtain a yellowish solution, which was then heated to 100 ℃ for reflux for 24 h to get colorless and transparent solution A. Solution A was dialyzed with deionized water for 24 h (water changed every 4 h), then dehydrated using a rotary evaporator (60 ℃ water bath), and finally dried at 140 ℃ for 10 h to obtain sample powder.

**Synthesis of GdVO_4_@Ce**

NaOH, GdCl_3_, ReCl_3_ (RE=Pr, Ce, Dy), EDTA-2Na and NaVO_3_ solutions were prepared separately. The NaVO_3_ aqueous solution was adjusted to pH~13 with 1 mol/L NaOH. Then 19.8 mL GdCl_3_, 1.87 mL EuCl_3_, 0.22 mL YCl_3_, 0.11 mL PrCl_3_, 16.5 mL EDTA-2Na and 16.5 mL Na_3_VO_4_ solutions were added into a round-bottomed flask, vigorously stirred for 10 min to obtain a yellowish solution, which was then heated to 100 ℃ for reflux for 24 h to get colorless and transparent solution A. Solution A was dialyzed with deionized water for 24 h (water changed every 4 h), then dehydrated using a rotary evaporator (60 ℃ water bath), and finally dried at 140 ℃ for 10 h to obtain sample powder.

**ROS scavenging activity by ESR spectra analysis and UV-Vis spectrum**

For the ESR assay, •OH were generated via the Fenton reaction using FeSO_4_·7H_2_O (0.9 mM) and H_2_O_2_ (4.2 mM). CeO_2_ and GVCP were separately introduced into the •OH solution, followed by the addition of DMPO as a spin trap. After 5 min, the ESR spectra were recorded to assess the radical-scavenging capacity of the three materials. The •OH elimination ability was also measured indirectly by a UV-Vis photometric method employing methyl viologen (MV). FeSO_4_·7H_2_O (0.15 mM), H_2_O_2_ (1 mM), MV (0.012 mM), and each material (200 μg mL⁻¹) were mixed in a total volume of 1 mL. Following incubation in the dark for 5 min, the ultraviolet absorption of the solution was recorded. A decrease in absorbance relative to the Fenton-only control indicated •OH clearance by the material. •O_2_^-^ scavenging was examined using an analogous procedure[1].

**Measurement of CAT-like activity**

The CAT-like activity of the nanozymes was assessed by monitoring the oxygen generated from H_2_O_2_ decomposition. In a typical assay, these formulations were mixed with H_2_O_2_ in 20 mL of Tris-HCl buffer (0.1 M, pH 7.52). The dissolved oxygen concentration (mg/L) was recorded over 30 min using a dissolved oxygen meter.

**Determination of PTE loading**

PTE was loaded onto GVC nanoparticles by mixing GVC with PTE at a predetermined GVC mass ratio under gentle stirring overnight at room temperature in the dark. The resulting GVCP nanoparticles were collected by centrifugation and washed to remove free PTE. The amount of unloaded PTE in the supernatant was quantified by HPLC based on a standard calibration curve of PTE. The drug loading content and encapsulation efficiency were calculated according to the following equations: drug loading content (%) = weight of loaded PTE/weight of GVCP × 100 %; encapsulation efficiency (%) = weight of loaded PTE/weight of initially added PTE × 100 %.

**DFT calculations**

All GaVO₄ and rare-earth-substituted (Ce, Dy, Pr) models were performed using spin-polarized density functional theory (DFT) as implemented in the Vienna Ab initio Simulation Package (VASP 5.4.4)[2, 3]. The exchange–correlation interactions were described by the generalized gradient approximation (GGA) with the Perdew–Burke–Ernzerhof (PBE) functional[4]. The ion-electron interactions were treated using the projector augmented wave (PAW) method[5]. A plane-wave cutoff energy of 450 eV was employed. The DFT+U approach was adopted to account for the strong Coulomb interactions among the transition-metal d electrons, with U parameters set to 3.0 eV for V and 4.0 eV for Gd, Ce, Dy, and Pr. Rare-earth pseudopotentials including f orbitals were utilized. All atomic positions were fully relaxed until the maximum force on each atom was less than 0.01 eV/Å. During structural relaxation, the Brillouin zone was sampled using a Monkhorst–Pack scheme with a 3 × 3 × 1 k‑point mesh centered at the Γ point. Van der Waals interactions were considered via the semiempirical DFT‑D3 approach[6]. A vacuum layer of approximately 15 Å was applied to prevent interactions between adjacent layers. All visualized structural data were rendered using VESTA software. The differential charge density, band structure, and electron localization function (ELF) analyses were carried out using the VASPKIT 1.3.5 tool[7].

**Isolation and Culture of Chondrocytes**

Primary chondrocytes were isolated from the articular cartilage of knee joints obtained from newborn (5-6 days old) male Sprague‑Dawley (SD) rats. Briefly, the cartilage tissue was minced into small fragments and digested with 0.2% type II collagenase for 5 h at 37 °C. The digested suspension was subsequently centrifuged at 350 × g for 10 min to pellet the cells. The collected chondrocytes were resuspended in complete culture medium, composed of 89% Dulbecco’s Modified Eagle Medium (DMEM), 10% fetal bovine serum (FBS), and 1% penicillin–streptomycin, and then seeded into T25 flasks. Cells were maintained in a humidified incubator at 37 °C under 5% CO_2_. Chondrocytes at passage 2 were used for all experiments. To establish an *in vitro* OA model, chondrocytes were treated with 20 ng/mL interleukin‑1β (IL‑1β) for 12 h.

**Assessment of Intracellular ROS Levels**

To evaluate the effect of GVCP on intracellular ROS, primary chondrocytes were divided into five groups: normal control, H_2_O_2_, GVC, PTE, and GVCP. The normal control group received no H_2_O_2_ treatment. Cells in the H_2_O_2_ group were exposed to H_2_O_2_ for 24 h. In the GVC, PTE, and GVCP groups, cells were first treated with H_2_O_2_ for 12 h, followed by incubation with GVC, PTE, or GVCP, respectively, for an additional 12 h. For ROS detection, cells from each group were washed three times with PBS and then incubated with DCFH‑DA (1:1000 dilution; S0033, Beyotime Biotechnology, China) for 20 min at 37 °C. Fluorescence images were captured using a confocal microscope, and the mean fluorescence intensity was quantified with Image J software.

**Quantification of RT‐PCR**

To assess the expression of target genes, total RNA was first isolated with TRIzol reagent. Subsequent reverse transcription of mRNA into complementary DNA (cDNA) was carried out using the Hifair II 1st Strand cDNA Synthesis Kit. Quantitative PCR was then performed with the Hieff qPCR SYBR Green Master Mix. The primers were designed to amplify COL2A1, ACAN, ADAMTS-5, SXO-9, COX-2, and MMP13, and their sequences are provided in Table S1 (Supporting Information). GAPDH was used as the internal reference gene, and relative gene expression levels were calculated *via* the ΔΔCt method.

**Western Blotting Analysis**

Total protein was extracted from chondrocytes in each group by cell lysis. The protein samples were then separated *via* SDS-PAGE and transferred onto PVDF membranes. Following transfer, the membranes were blocked with 5% non‑fat dry milk. Subsequently, they were incubated overnight at 4°C with specific primary antibodies diluted as follows: anti‑GAPDH (1:10,000), anti‑Col II (1:1,000), anti‑MMP13 (1:1,000), anti‑COX2 (1:1,500), anti‑SOX9 (1:1,000), anti‑ACAN (1:1,000), anti‑BCL‑2 (1:1,000), anti‑BAX (1:1,000), and anti‑ADAMTS-5 (1:250). After thorough washing, the membranes were probed with an HRP-conjugated secondary antibody for 1 hour at room temperature. Protein bands were finally detected using an enhanced chemiluminescence (ECL) substrate and imaging system.

**Immunofluorescence Staining**

Following treatment, the chondrocytes or RAW264.7 cells were washed three times with PBS. They were then fixed with 4% paraformaldehyde for 20 min, permeabilized with 0.5% Triton X-100 for 15 minutes, and blocked with 5% serum. Subsequently, the cells were incubated overnight at 4°C with primary antibodies specific for the following targets: Col II (1:200), SOX-9 (1:200), ACAN (1:150), MMP13 (1:100), BCL-2 (1:100), COX-2 (1:100), CD86 (1:200), iNOS (1:200), CD206 (1:200), and Arg-1 (1:200). After washing, the cells were incubated with Alexa Fluor 488- or Alexa Fluor 594-conjugated secondary antibodies. for 1 hour at room temperature. Finally, the cells were counterstained with FITC-labeled phalloidin and DAPI and visualized using a confocal laser scanning microscope.

**Surgical OA Model in Rats**

The OA model mimicking advanced human disease was generated in male Sprague‑Dawley rats (250-300 g) *via* surgical destabilization of the knee. This was achieved by transecting the anterior cruciate ligament (ACLT) in conjunction with partial removal of the medial meniscus (pMMx). The surgical protocol was as follows[8]: after standard anesthetic preparation, the right hindlimb was shaved and sterilized. An initial medial parapatellar skin incision provided access to the knee region. A subsequent longitudinal cut then allowed careful entry into the joint capsule. To achieve sufficient exposure, the patella was shifted laterally and the underlying fat pad was excised. Rats assigned to the sham group underwent no further intervention at this point. For animals in the OA model groups (ACLT + pMMx), the medial meniscotibial ligament (MMTL) was first severed, followed by transection of the central portion of the medial meniscus, using specialized micro-dissection tools. Next, the anterior cruciate ligament was completely transected. Successful induction of joint instability was verified by a positive anterior drawer sign. Upon completion of the ligament and meniscal procedures, the patella was returned to its anatomical position. The surgical site was then closed through layered suturing of the capsule and skin. Postoperatively, rats were administered antibiotics and analgesics and were maintained in single cages. Animals were randomly assigned to five experimental groups: (1) Sham surgery with PBS injection (Sham), (2) ACLT + pMMx with PBS (PBS), (3) ACLT + pMMx with free PTE (PTE), (4) ACLT + pMMx with GVC (GVC), and (5) ACLT + pMMx with GVCP nanosystem (GVCP). These formulations were first administered *via* intra‑articular injection into the operated knee two days post‑surgery. At the 4-week endpoint, rats were euthanized, and knee joints, major organs, and whole-blood samples were collected for the indicated analyses. At the 8-week endpoint, the remaining rats were euthanized, and their knee joints were collected to evaluate the sustained therapeutic effects.

**Footprint Analysis**

Post-intervention gait performance was evaluated using an automated gait analysis system (CatWalk XT, model BT60101, Zhongshi Scientific). After complete recovery, animals were acclimatized to the glass walkway through twice‑daily 10‑minute habituation sessions over five consecutive days. During the formal test, rats were allowed to traverse the runway voluntarily, and dynamic footprint data were continuously recorded by the system. The following parameters were automatically derived: stride length, swing cycle, duty cycle, and forelimb/hindlimb paw print patterns.

**Micro‐Computed Tomography**

Following a one-month treatment period, the knee joints were harvested and dissected free of surrounding soft tissues. The specimens were fixed in formalin for 48 hours. Subsequently, micro‑CT scanning was conducted using an Inveon system (Siemens, Germany) with the following parameters: 55 kV X‑ray voltage, 145 μA current, and an integration time of 314 ms. Three‑dimensional reconstruction and histomorphometric analysis of the tibial subchondral bone were performed using the Inveon Research Workplace software. Parameters quantified from coronal images included the bone volume fraction (BV/TV), trabecular number (Tb.N), trabecular thickness (Tb.Th), trabecular separation (Tb.Sp), and trabecular pattern factor (Tb.Pf).

**Scanning electron microscope observation**

The ultrastructure and elemental composition of the cartilage surface were analyzed using a field-emission scanning electron microscope (FE-SEM, Hitachi S-4800) integrated with an energy-dispersive X-ray spectrometer (EDS).

**Histological and Immunohistochemistry Staining and Analysis**

Whole rat knee joints were fixed in 4% neutral buffered formalin for 48 hours, followed by decalcification in EDTA solution for 2 weeks. The specimens were then dehydrated through a graded ethanol series, cleared in xylene, and embedded in paraffin. Serial sagittal sections (5 µm in thickness) were cut through the medial compartment of the knee joint. The sections were subjected to various staining protocols: TB for proteoglycan visualization, SO&FG, and H&E. For immunohistochemical analysis, sections were incubated with primary antibodies against SOX9, COL 2, COX-2, BCL-2, Bax, and p53. Histopathological changes in articular cartilage and synovium were evaluated according to the OARSI scoring system[9]. Quantitative analysis was performed on captured images using ImageJ software by an investigator blinded to the experimental groups. Furthermore, the severity of synovitis was graded based on H&E-stained sections to assess joint inflammation.

**Statistical Analysis**

Data are presented as mean ± SD. Statistical analysis was performed using one-way or two-way ANOVA followed by Tukey’s post hoc test in GraphPad Prism 8.0. Significance levels were set as **p* < 0.05, ***p* < 0.01, ****p* < 0.001, and *****p* < 0.0001; “ns” indicates no statistically significant difference.

**Figure S1** The SOD-like and CAT-like activities of the indicated formulations. (A) The SOD-like activity, reflecting the capacity to scavenge •O_2_^-^. (B) The CAT-like activity, reflecting the capacity to catalyze H_2_O_2_ decomposition. Data are presented as mean ± SD. **p* < 0.05, ***p* < 0.01, ****p* < 0.001, and *****p* < 0.0001.

**Figure S2.** Fourier transform infrared (FTIR) spectra of PTE and GVCP.

**Figure S3**. Cell-free DPPH radical-scavenging activity of different formulations. (A) Representative photographs of DPPH solutions after incubation with PBS (G1), GVC (G2), free PTE (G3), a physical mixture of GVC and PTE (G4), or GVCP (G5). Progressive discoloration of the DPPH solution indicates increasing radical-scavenging activity. (B) Quantification of the DPPH radical-scavenging rate. GVCP exhibited the highest scavenging activity and outperformed the corresponding physical mixture of GVC and PTE. Data are presented as mean ± SD, n=6. *****P* < 0.0001.

**Figure S4.** Representative fluorescence micrographs from day 2 showing live/dead staining of the control, GVC, PTE, and GVCP groups. Scale bar, 50 μm.

**Figure S5.** Quantitative analysis of DFCHA. Data are presented as mean ± SD (n = 6). **p* < 0.05, ***p* < 0.01, ****p* < 0.001 and *****p* < 0.0001.

**Figure S6.** GVCP suppresses LPS-induced pro-inflammatory cytokine expression in macrophages. (A) Representative immunofluorescence images of IL-6 (red) and TNF-α (green) in RAW264.7 macrophages. (B-C) Quantification of the relative fluorescence intensities of IL-6 (B) and TNF-α (C). Data are presented as mean ± SD, n=6. **P* < 0.05, ***P* < 0.01, and *****P* < 0.0001. Scale bar, 50 μm.

**Figure S7.** GV promotes the expression of M2-associated macrophage markers. (A) Representative immunofluorescence images of CD206 (red, upper row) and Arg-1 (red, lower row) in macrophages treated with control medium (G1), GV (G2), GVC (G3), or GVCP (G4). (B-C) Quantification of the relative fluorescence intensities of CD206 (B) and Arg-1 (C). GV treatment increased the expression of both M2-associated markers compared with the control, supporting the intrinsic ability of GV to regulate macrophage M2 polarization. GVC and GVCP elicited further increases, with GVCP producing the most pronounced response. Scale bar:50 μm. Data are presented as mean ± SD. ****P* < 0.001 and *****P* < 0.0001.

**Figure S8.** Gd^3+^ contributes to GVCP-induced M2 macrophage polarization. A-B. Representative immunofluorescence images of CD206 (A) and Arg-1 (B) in RAW264.7 macrophages. C-D. Quantitative analysis of CD206 (C) and Arg-1 (D) fluorescence intensity. RAW264.7 macrophages were treated with control medium (G1), GVCP (G2), GVCP + DOTA (G3), or GdCl_3_ (G4). GVCP significantly increased CD206 and Arg-1 expression, whereas DOTA-mediated Gd^3+^ chelation markedly reduced this effect. GdCl_3_ alone partially promoted CD206 and Arg-1 expression. Data are presented as mean ± SD (One-way ANOVA with Tukey’s post hoc for multi-group analysis). **p* < 0.05, ***p* < 0.01, ****p* < 0.001, and *****p* < 0.0001.

**Figure S9.** Quantitative analysis of ACAN in primary chondrocytes.

**Figure S10.** The chondrocyte-protective effect of GVCP. A. Representative IF images of BAX in primary chondrocytes. B. Quantitative analysis of BAX in primary chondrocytes. Scale bar: 50μm. Data are presented as mean ± SD (n = 6). **p* < 0.05, ***p* < 0.01, ****p* < 0.001 and *****p* < 0.0001.

**Figure S11.** A. Representative western blot images of Adamts-5, COX-2, SOX-9, BCL-2, BAX, MMP-13, COL II, and ACAN in chondrocytes. GAPDH was used as a loading control. B. Quantitative analysis of Adamts-5, COX-2, SOX-9, BCL-2, BAX, MMP-13, COL II and ACAN protein expression levels from (A). Data are presented as mean ± SD (n = 6). **p* < 0.05, ***p* < 0.01, ****p* < 0.001 and *****p* < 0.0001.

**Figure S12.** A. Representative Western blot images of p53, Bcl-2, Bax, and GAPDH in IL-1β-stimulated chondrocytes. B-D. Quantitative analysis of p53 (B), Bcl-2 (C), and Bax (D) expression. GAPDH was used as the loading control. G1: normal control; G2: IL-1β; G3: IL-1β + C16-Ceramide; G4: IL-1β + GVCP; G5: IL-1β + GVCP + C16-Ceramide. Data are presented as mean ± SD (One-way ANOVA with Tukey’s post hoc for multi-group analysis). **p* < 0.05, ***p* < 0.01, ****p* < 0.001, and *****p* < 0.0001.

**Figure S13.** GVCP inhibits osteophyte formation and reverses pathological subchondral bone remodeling. A. representative micro-CT images showing 2D coronal slices (top) and 3D joint reconstructions (bottom) with osteophytes marked by red arrows. B. Quantitative micro-CT analysis of tibial subchondral bone parameters (Tb.Th, Tb.Pf, BV/TV, Tb.Sp, Tb.N; n=3). Data are presented as mean ± SD (One-wayANOVA with Tukey’s post hoc for multi-group analysis). **p* < 0.05, ***p* < 0.01, ****p* < 0.001, and *****p* < 0.0001.

**Figure S14.** A-D Representative histological images of (A) SO&FG, (B) TB, and (C-D) H&E staining of knee joints from sham- or ACLT+pMMx-operated rats after 8‑week treatment (scale bars: 200 μm). E-F. Corresponding semiquantitative scores for cartilage degeneration and synovitis. Data are presented as mean ± SD (One-wayANOVA with Tukey’s post hoc for multi-group analysis). **p* < 0.05, ***p* < 0.01, ****p* < 0.001, and *****p* < 0.0001.

**Figure S15**. Schematic illustration of the therapeutic mechanism of GVCP against OA. GVCP functions as a nanozyme to scavenge ROS via SOD- and CAT-mimicking activities, while sustaining PTE release to restore mitochondrial homeostasis, thereby inhibiting inflammation and attenuating OA progression.

**Figure S16.** Representative histology of major organs following treatment. Histological evaluation revealed no pathological alterations in any treatment group compared to controls. Scale bar, 100 μm.

**Figure S17.** Hematological analysis post-treatment. The results of routine blood tests are expressed as mean ± SD, with n = 3 per group. Statistical comparison indicated no significant differences between any of the groups.

**Figure S18.** Assessment of blood biochemical markers. Serum parameters were measured to evaluate (A) renal function and (B) liver function. All data are shown as mean ± SD (n = 3), with no statistically significant differences detected between groups.

**Table S1.** Primer sequences of the genes for qRT-PCR analysis

| Gene | Forward primer (5’-3’) | Reverse primer (5’-3’) |
| --- | --- | --- |
| GAPDH | GAAGAAGGTGGTGAAGCAGGG | CACTGTTGAAGTCGCAGGAG |
| Col 2a1 | CACGCTCAAGTCCCTCAACAG | TCTATCCAGTAGTCACCGCTCT |
| ACAN | GGAGGAGCAGGAGTTTGTCAAA | TGTCCATCCGACCAGCGAAAAC |
| ADAMT5 | GTCCAAATGCACTTCAGCCACGAT | AATGTCAAGTTGCACTGCTGGGTG |
| MMP13 | TTCTGGTCTTCTGGCACACG | TGGAGCTGCTTGTCCAGGT |
| SOX-9 | GCAAACACGTTGCAAATGGC | AAGTCCAAACAGGCAGGGAG |
| COX-2 | GATGTTCGCATTCTTTGCCCA | TACACCTCTCCACCGATGAC |

**Reference**

[1] K. Song, F. Chen, M. Li, Q. Gao, X. Zhu, W. Liu, S. Guan, J. Han, Eu-doping engineered CoAl-layered double hydroxides for efficient reactive oxygen species scavenging, Chemical Engineering Journal 521 (2025) 166555. <https://doi.org/https://doi.org/10.1016/j.cej.2025.166555>.

[2] G. Kresse, J. Hafner, Ab initio molecular dynamics for open-shell transition metals, Phys Rev B Condens Matter 48(17) (1993) 13115-13118. <https://doi.org/10.1103/physrevb.48.13115>.

[3] G. Kresse, J. Hafner, Ab initio molecular dynamics for liquid metals, Physical Review B 47(1) (1993) 558-561. <https://doi.org/10.1103/PhysRevB.47.558>.

[4] J.P. Perdew, K. Burke, M. Ernzerhof, Generalized Gradient Approximation Made Simple, Physical Review Letters 77(18) (1996) 3865-3868. <https://doi.org/10.1103/PhysRevLett.77.3865>.

[5] G. Kresse, D. Joubert, From ultrasoft pseudopotentials to the projector augmented-wave method, Physical Review B 59(3) (1999) 1758-1775. <https://doi.org/10.1103/PhysRevB.59.1758>.

[6] S. Grimme, J. Antony, S. Ehrlich, H. Krieg, A consistent and accurate ab initio parametrization of density functional dispersion correction (DFT-D) for the 94 elements H-Pu, The Journal of Chemical Physics 132(15) (2010). <https://doi.org/10.1063/1.3382344>.

[7] V. Wang, N. Xu, J.-C. Liu, G. Tang, W.-T. Geng, VASPKIT: A user-friendly interface facilitating high-throughput computing and analysis using VASP code, Computer Physics Communications 267 (2021) 108033. <https://doi.org/https://doi.org/10.1016/j.cpc.2021.108033>.

[8] B.C. Geiger, S. Wang, R.F. Padera, Jr., A.J. Grodzinsky, P.T. Hammond, Cartilage-penetrating nanocarriers improve delivery and efficacy of growth factor treatment of osteoarthritis, Sci Transl Med 10(469) (2018). <https://doi.org/10.1126/scitranslmed.aat8800>.

[9] N. Gerwin, A.M. Bendele, S. Glasson, C.S. Carlson, The OARSI histopathology initiative - recommendations for histological assessments of osteoarthritis in the rat, Osteoarthritis Cartilage 18 Suppl 3 (2010) S24-34. <https://doi.org/10.1016/j.joca.2010.05.030>.
